# Burden of Endometriosis: Infertility, Comorbidities, and Healthcare Resource Utilization

**DOI:** 10.3390/jcm11041133

**Published:** 2022-02-21

**Authors:** Vered H. Eisenberg, Dean H. Decter, Gabriel Chodick, Varda Shalev, Clara Weil

**Affiliations:** 1 Sheba Medeical Center, Sackler Faculty of Medicine, Tel Aviv University, Tel Aviv 6997801, Israel; deanhdecter@gmail.com; 2 Maccabi Institute for Research and Innovation, Maccabi Healthcare Services, Sackler Faculty of Medicine, Tel Aviv University, Tel Aviv 6997801, Israel; vered.eisenberg@moh.gov.il (G.C.); deandecter@mail.tau.ac.il (V.S.); d.decter@yahoo.com (C.W.)

**Keywords:** endometriosis, infertility, co-morbidities, burden, healthcare resource utilization, real world data, epidemiology, adolescents, young adults

## Abstract

The goal of our study was to evaluate the burden of endometriosis in the community by comparing healthcare resource utilization, total direct medical costs, infertility, and comorbidity rates of women with and without a diagnosis of endometriosis. A retrospective case–control study was performed using the databases of a 2.1 million-member nationwide healthcare plan. The study population included women aged 15–55 years enrolled in the healthcare plan. Women with a diagnosis (ICD-9) of endometriosis were compared to controls without diagnosed endometriosis. Women were individually matched (1:4) on age and residence area. Patient characteristics were described, including infertility, comorbidities, and annual healthcare resource utilization. Total direct medical costs were analyzed in a generalized linear model adjusting for age. Women with endometriosis (*n* = 6146, mean age ± SD: 40.4 ± 8.0 y) were significantly more likely than controls (*n* = 24,572) to have a lower BMI and a higher socioeconomic status. After adjusting for BMI and socioeconomic status, endometriosis was significantly associated with infertility (OR = 3.3; 95% CI 3.1–3.5), chronic comorbidities, higher utilization of healthcare services (hospitalization: OR = 2.3; 95% CI 2.1–2.5), pain medications, and antidepressants. Women aged 15–19 y with endometriosis had substantially higher utilization of primary care visits (57.7% vs. 14.4%) and oral contraceptive use (76.9% vs. 9.6%). Direct medical costs associated with endometriosis were higher than those for controls (OR = 1.75; 95% CI 1.69–1.85). Endometriosis is associated with a high burden of comorbidities, increased healthcare resource utilization, and excess costs, particularly for younger patients whose healthcare needs may differ widely from the older population.

## 1. Introduction

Endometriosis is a benign gynecological condition defined as the presence of endometrial-like glands and stroma outside of the uterus [1]. The prevalence of endometriosis is approximately 10% [2,3]; however, estimates differ widely due to varying study populations and designs [4]. In line with population-based estimates from European databases (range 0.8–1.8%) [5,6], the prevalence of diagnosed endometriosis has been reported as 10.8 per 1000 (95% CI 10.5–11.0) [7], which is lower than estimates based on high-risk populations.

Symptoms of endometriosis include chronic pelvic pain, dysmenorrhea, dyspareunia, dysuria, dyschezia, and subfertility [1,3]. Symptoms may appear years before diagnosis [8], resulting in an average 10-year delay [2,6,9]. The gold standard for diagnosis includes laparoscopy with or without biopsy; however, there is support for treating symptoms prior to definitive surgical diagnosis [10]. There are arguments for shifting diagnosis away from surgical and more towards clinical aims to focus more on the patient and less on the lesion to reduce the cost and diagnostic delay [10,11,12]. Pain-relieving and hormonal medications are frequently used in treatment [13]. Endometriosis patients may pay an average of seven visits [2] to a general practitioner before being referred to a specialist and may undergo symptom treatment without a confirmed diagnosis [14], contributing to the burden on healthcare resources. 

There are relatively few studies on endometriosis in adolescent women [15]. Little is known about this disease in adolescent women, and an earlier diagnosis may improve outcomes to reduce chronic pain and improve quality of life. The present study aimed to assess the burden of endometriosis by comparing healthcare resource utilization (HCRU), total direct medical costs, infertility, and comorbidity rates of women with and without an endometriosis diagnosis. 

## 2. Materials and Methods

We conducted a retrospective population-based case–control study using the computerized databases of the local healthcare service. The database includes approximately 2 million active members nationwide and represents 25% of the national population with similar sociodemographic characteristics [16]. Data sources are described in detail in another publication [7].

Women with endometriosis were identified according to diagnosis codes (International Classification of Disease, 9th Edition, clinical modification (ICD-9): 617.xx) from a primary care physician (PCP) (general practitioner, pediatrician, gynecologist), or another specialist from 1998 to 2015. The study population consisted of women aged 15–55 years on 31 December 2015, with diagnosed endometriosis and at least 12 months of continuous enrollment in the health plan. While the results of imaging and surgical evaluation were not used for validating the diagnosis, more than 90% had a previous record of a pelvic/gynecological US examination. This population and the validity of case ascertainment methods are described in detail in another study [7]. For comparison of patient characteristics and healthcare resource utilization, controls were selected from the database’s general population with no record of endometriosis and individually matched (1:4) on age (5-year groups) and residential area. 

Data were obtained on patients’ age, region of residence (Northern/Center/Southern regions), and socioeconomic status (SES). SES was based on a commercial geographic index (range: 1–10) developed by Points Ltd., which is correlated with the residence-based SES from the local Central Bureau of Statistics [17]. SES was classified into low (1–3), medium (4–6), and high (7–10). Body mass index (BMI) categories were based on WHO recommended cut-off points [18]. 

Broad coverage of infertility assistance is provided in the National Basket of Health Services. Data were extracted from the healthcare database infertility registry which integrates data on subfertility or infertility diagnoses, fertility treatments in hospital or community clinics (including in vitro fertilization, ovarian stimulation procedures, and oocyte donation), and dispensed fertility medications [19]. 

Data on chronic comorbidities were obtained from previously validated registries for cardiovascular disease (CVD) [20], diabetes [21], hypertension [22], and chronic kidney disease (CKD) [23], and from the National Cancer Registry [24]. The Deyo–Charlson Comorbidity Index (CCI) [25], based on ICD-9 codes, was modified to include additional data from the healthcare service chronic disease registries. Smoking status was obtained from physician reports. Inflammatory bowel disease (IBD) was described using ICD-9 codes. We described diagnoses of conditions with potentially overlapping symptoms, which may be associated with or misdiagnosed as endometriosis, including appendicitis, irritable bowel syndrome (IBS), and unspecified bladder disorders. 

To evaluate HCRU, the frequency of hospitalization and visits to PCPs and gynecologists was described. Use of pain medications was defined as at least 2 dispensed prescriptions in one year, according to the following categories: nonsteroidal anti-inflammatory drugs (NSAIDs); paracetamol (PAR), acetylsalicylic acid (ASA), or dipyrone; codeine combined with either PAR or ASA; tramadol. Use of antidepressants was defined as at least 2 dispensed prescriptions of selective serotonin reuptake inhibitors, or tricyclic or other antidepressants. Data were also obtained on dispensed oral contraceptives, gonadotropin medication, and insertion of hormone-releasing intrauterine devices (IUDs). Laboratory testing rates were described for complete blood tests (CBC), serum iron, luteinizing hormone (LH), follicle-stimulating hormone (FSH), and cancer antigens (CA-125 and CA-15.3). Utilization of magnetic resonance imaging (MRI) and transvaginal ultrasound (TVUS), including utilization of a dedicated endometriosis TVUS (E-TVUS), was described. The impact on work productivity, school attendance, and performance of daily activities [2] was not within the scope of the current study. 

Total annual direct medical costs were obtained from the local healthcare service database, as described in previous cost-of-illness studies [26]. Briefly, most services in the local healthcare service are delivered via third-party providers, and annual costs are routinely calculated in the accounting system as the sum of all services provided, including per diem hospitalization rates, physician visit reimbursements, emergency room visits, laboratory tests, procedures, and hospital outpatient charges. Out-of-pocket expenses and services provided directly by the state, such as hospital birthing costs and certain mental healthcare services, were not captured. Costs were translated into cost units to avoid disclosure of internal corporate information.

Patient characteristics: Descriptive statistics were generated for analysis variables, including frequency distributions for categorical variables (*n*; %), and mean values with standard deviations (SD) or medians with interquartile ranges (IQRs) for continuous variables. Differences between groups were tested using the χ^2^ test, t-test, or median test. Odds ratios (ORs) and 95% confidence intervals (CIs) were computed in a conditional logistic regression, adjusting for SES and BMI. A generalized linear model (gamma distribution with log link) with log-transformed cost, adjusting for age (cubic), was used to estimate predicted costs in both groups and to calculate the adjusted OR (95% CI) associated with endometriosis. Analyses were performed with IBM-SPSS version 24 (IBM Corp. Released 2013. IBM SPSS Statistics for Windows, Version 22.0. Armonk, NY, USA: IBM Corp.). 

The study was approved by the institutional review board (reference number 2016027). Written informed consent was not required as this was a retrospective database analysis. 

## 3. Results

A total of 6146 women aged 15–55 were included in the endometriosis prevalence population (mean age ± SD: 40.4 ± 8.0), and 24,572 women were included in the matched control group. Compared to controls, women with endometriosis were significantly more likely to have a higher SES and a lower BMI and reside in the Central region, and less likely to live in areas with predominantly religious orthodox Haredi or Arab populations (Table 1). 

**Figure 1:**
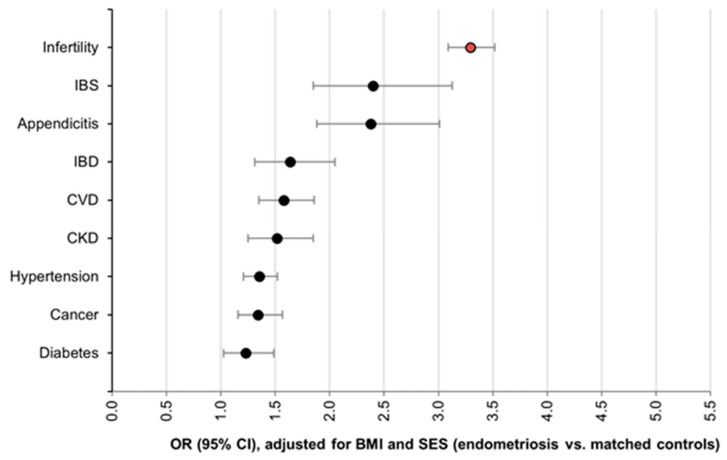
Odds ratios for comorbidities among women diagnosed with endometriosis compared to matched controls, adjusted for SES and BMI (lifetime prevalence).

Infertility was present in 36.9% of women with endometriosis (Table 2), corresponding to an OR of 3.3 (95% CI: 3.1–3.5), adjusting for SES and BMI (Figure 1). Women aged 40–44 had the highest lifetime prevalence of infertility (49.9% vs. 21.7%). Chronic comorbidities (CVD, hypertension, diabetes, cancer, and CKD) were significantly more prevalent among women with endometriosis, with ORs ranging from 1.2 to 1.6 (Figure 1). Women with endometriosis also had a significantly higher prevalence of irritable bowel syndrome and appendicitis—conditions with potentially overlapping abdominal pain symptoms, where the distinction between comorbidity and misdiagnosis is often challenging (Table 2 and Figure 1). 

### 3.1. Healthcare Resource Utilization and Direct Medical Costs

Women with endometriosis were significantly more likely than controls to have seen a gynecologist or other PCP within the year. Almost 20% of endometriosis patients visited a gynecologist at least 5 times during the year, 1.6 times as high as controls (*p* < 0.05 for adjusted OR). Significantly higher annual rates of admission for hospitalization or ER visits were observed among endometriosis patients compared to controls. Women with endometriosis were more than twice as likely to have been hospitalized at least once, and three times as likely to have been hospitalized at least twice. ORs adjusted for SES and BMI are presented in Table 3. 

**Figure 2:**
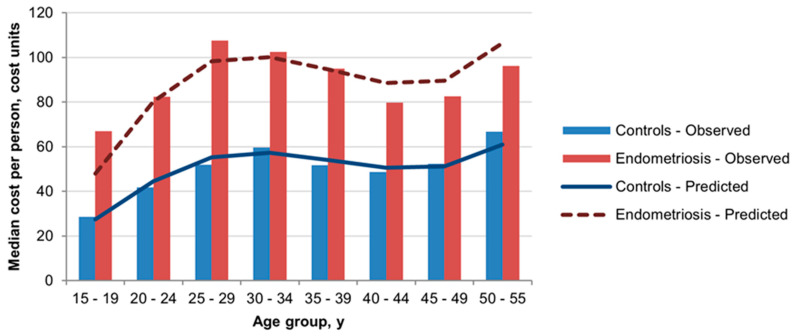
Observed and predicted median costs per person (endometriosis vs. controls) based on total annual direct medical costs in the local healthcare service and estimates from the generalized linear model adjusted for age.

Oral contraceptive use was 23.6% (vs. 15.6%) in the past year, and 71.0% (vs. 53.2%) for lifetime use. Women aged 25–29 had the highest rates of oral contraceptive use in 2015: 86.4% and 73.4% among endometriosis patients and controls, respectively. The annual rate of insertion of a hormone-releasing IUD was similar in the two groups and was low overall. Gonadotropin use was three times as high among endometriosis patients compared to controls (Table 3). Pain medication use was significantly higher in the endometriosis group, particularly for narcotic-like medications (tramadol). In addition, 11.8% of women with endometriosis used antidepressants in the past year (vs. 8.6% of controls). Adjusted ORs are presented in Table 3. Utilization of imaging procedures (MRI and US) was increased in the endometriosis group compared to controls. Overall, 42.2% of women with endometriosis had a pelvic/genital US, of which only 3.1% underwent a dedicated endometriosis US (Table 3). Compared to controls, endometriosis patients were significantly more likely to have had CBC and LH or FSH testing. CA-125 and CA-15.3 testing was associated with adjusted ORs (95% CI) of 5.9 (5.2–6.7) and 3.2 (2.6–3.9), respectively. Overall, endometriosis was associated with an excess in total per-person direct medical costs across all age groups (Figure 2) and with an age-adjusted OR of 1.75 (95% CI 1.69–1.85).

### 3.2. Burden among Adolescents and Young Women

In the youngest age group, 15–19 years, women with endometriosis (*n* = 52) were significantly (*p* < 0.05) more likely than controls (*n* = 208) to have seen a PCP (76.9% vs. 46.2%) and/or gynecologist (57.7% vs. 14.4%), and to have used oral contraceptives (76.9% vs. 9.6%). Among women aged 20–24 y, smaller but significant differences were also seen in PCP (92.8% vs. 83.1%) and specifically gynecologist (76.6% vs. 54.8%) visit rates and use of oral contraceptives (83.8% vs. 56.7%). Use of pain medication was more likely among endometriosis patients than controls, for the age groups 15–19 y (19.2% vs. 3.8%) and 20–24 y (15.6% vs. 5.7%). Among women aged 20–24, endometriosis was also associated with significantly higher antidepressant use (7.8% vs. 3.4%). Median direct medical costs per person for endometriosis were twice those of controls in the youngest age groups (Figure 2). 

## 4. Discussion

The results of this study indicate that endometriosis was significantly associated with a higher burden of infertility, chronic comorbidities, utilization of healthcare services, pain medications, and antidepressants, and overall, 1.75-fold higher direct medical costs. The excess burden among young women aged 15–24 years reflects substantially higher utilization of gynecologist visits and oral contraceptives. 

Patients diagnosed with endometriosis had higher HCRU than controls in all age groups. This is consistent with previous reports and is also associated with a higher economic and societal burden [2,27,28]. A meta-analysis of 24 studies showed an association between endometriosis and depressive symptoms which was mostly determined by chronic pelvic pain [29]. Patients with endometriosis are more likely to have associated comorbidities than controls. Previous studies [30,31,32,33,34] reported that women with endometriosis were more likely to have breast or ovarian cancer, autoimmune disease, or risk factors for cardiovascular disease. Fibromyalgia, interstitial cystitis, and IBD are also more commonly reported, possibly because of overlapping pain symptoms, resulting in a false or delayed diagnosis of endometriosis [35,36]. The co-occurrence of endometriosis and fibromyalgia is associated with an increased risk of anxiety or depression and HCRU [37]. We previously found a 10-fold increase in deep infiltrative endometriosis in patients with GI symptoms, suggesting the need to rule out endometriosis prior to performing any invasive GI procedures [38]. Our study found that women with endometriosis were more likely to have a lower BMI, and this inverse relationship has been demonstrated previously [39]. However, it should be mentioned that others have noted that the occurrence of obesity in women with endometriosis undergoing surgery was not a rare occurrence (7.4%) [40]. 

The heterogeneity of HCRU in endometriosis patients is also influenced by the development of subsequent comorbidities [41]. Increasing awareness may improve prevention and early detection. This is particularly true for the younger patient population since their pain-related symptoms may eventually result in infertility [42], incurring additional costs [43]. Fuldeore et al. speculated that older women may be more accustomed to or tolerant of painful symptoms over time and therefore reported less often to a healthcare facility [31]. This is plausible but may be less apparent in our population as gynecologist visits were more frequent in women with endometriosis compared to controls across all age groups. High rates of HCRU may also occur in cases of delayed diagnosis of endometriosis. In one retrospective analysis, over a third of women were referred for repeated consultations, and ultrasound was helpful in diagnosing endometriosis in only 10.6% of women [44]. However, inexperienced investigators may yield false negative results, providing false reassurance, and contributing to the diagnostic delay. 

In this study, we found a specific increase in HCRU in the younger population, similar to previous work [15]. It has been reported that 20–40% of adolescents reported missing school or declining performance and concentration due to their pelvic symptoms [45,46] as well as increased comorbid anxiety and depression [47]. The negative impact that chronic pain has on mental health may explain the increased use of antidepressants. Awareness of this association between dysmenorrhea and mental health in adolescents is warranted. Earlier diagnosis in the adolescent community may reduce the level of depression and improve quality of life [47]. Additionally, earlier diagnosis may also influence HCRU in this population for better or worse. For example, local excision of endometriosis lesions has been associated with higher rates of reoperation, whereas hysterectomy has been associated with lower reoperation rates [48].

Our clinical setup is based on universal health coverage with high access to PCPs and treatment. The main strength of this study is the large, validated population-based database representing an average-risk population, rather than symptomatic patients at dedicated endometriosis or infertility clinics. For these reasons, our findings related to clinical characteristics and comorbidities may be generalizable to similar populations worldwide. Previous studies evaluating the impact of individual comorbidities on healthcare spending have relied on claims analysis only [41].

There are several limitations in this retrospective database study. Case ascertainment was based entirely on physician codes, and patients were included without confirmation by surgery or imaging. Most patients in this setting would be followed up in the community without surgical intervention. Some patients may not have been captured if the physician did not enter the diagnosis, in which case the actual prevalence may be higher, as previously discussed [7]. 

HCRU may have been affected by treatment type and duration, and women in the control group may also include those with symptomatic but undiagnosed disease. This may lead to underestimation of the disparity in symptomatic burden between the groups. The likelihood that women are experiencing symptoms years prior to diagnosis results in an inherent limitation. The symptomatic occurrence may be greater than symptomatic diagnosis, potentially enabling some endometriosis patients to be included in the control group. Autoimmune diseases were defined according to at least one ICD-9 diagnosis code from any physician, so misclassification of suspected disease is possible [5,33,34]. Previous studies have shown that women diagnosed with endometriosis were more likely than controls to be diagnosed with IBS, IBD, or other comorbidities, and there may be overlapping symptoms [5,33,34,41], underscoring the challenge of distinguishing between misdiagnosis and comorbidity. 

In conclusion, women with a diagnosis of endometriosis have a significantly higher burden of infertility and chronic comorbidities, increased HCRU, and excess costs, particularly younger patients whose healthcare needs may differ widely from those of otherwise healthy women.

## Figures and Tables

**Table 1:** Sociodemographic characteristics and BMI of women diagnosed with endometriosis compared to matched controls.

Patient Characteristics ^1^ (31 December 2015)		Endometriosis (*n* = 6146)	Controls (*n* = 24,572)
Age in 2015, y (matched)	Mean ± SD	40.4 ± 8.0	40.4 ± 8.1
	15–19	52 (0.8%)	208 (0.8%)
	20–24	167 (2.7%)	668 (2.7%)
	25–29	428 (7.0%)	1712 (7.0%)
	30–34	722 (11.7%)	2888 (11.8%)
	35–39	1187 (19.3%)	4744 (19.3%)
	40–44	1620 (26.4%)	6472 (26.3%)
	45–49	1173 (19.1%)	4692 (19.1%)
	50–55	797 (13.0%)	3188 (13.0%)
Residence area (matched)	Central region	4143 (67.4%)	16,572 (67.4%)
	Northern	1104 (18.0%)	4416 (18.0%)
	Southern	896 (14.6%)	3584 (14.6%)
SES	Low (1–4)	833 (13.6%)	4058 (16.5%)
	Medium (5–6)	2239 (36.4%)	9189 (37.4%)
	High (7–10)	3059 (49.8%)	11,209 (45.6%)
	Missing	15 (0.2%)	116 (0.5%)
Population diversity	Haredi (Jewish orthodox)	157 (2.6%)	1223 (5.0%)
	Arab	245 (4.0%)	1268 (5.2%)
BMI	Mean ± SD	24.8 ± 5.2	25.5 ± 5.5
BMI category	<18.5	305 (5.0%)	853 (3.5%)
	18.5–25.0	2964 (48.2%)	10,553 (42.9%)
	25.0–30.0	1364 (22.2%)	5425 (22.1%)
	≥30.0	818 (13.3%)	3796 (15.4%)
	Missing	695 (11.3%)	3945 (16.1%)

^1^ All *p* values for unmatched variables are <0.001. SES, socioeconomic status; BMI, body mass index.

**Table 2:** Comorbidities among women diagnosed with endometriosis compared to matched controls.

Patient Characteristics (31 December 2015)		Endometriosis (*n* = 6146)	Controls (*n* = 24,572)
Infertility	All ages 15–55	2269 (36.9%)	3790 (15.4%)
	15–30	80 (12.4%)	92 (3.6%)
	30–34	218 (30.2%)	356 (12.3%)
	35–39	538 (45.3%)	866 (18.3%)
	40–44	808 (49.9%)	1403 (21.7%)
Conditions with overlapping symptoms	IBD	113 (1.8%)	262 (1.1%)
	IBS	92 (1.5%)	149 (0.6%)
	Appendicitis	114 (1.9%)	188 (0.8%)
Chronic comorbidities	CVD	222 (3.6%)	551 (2.2%)
	Hypertension	467 (7.6%)	1531 (6.2%)
	Diabetes	155 (2.5%)	550 (2.2%)
	Cancer	240 (3.9%)	703 (2.9%)
	CKD	144 (2.3%)	389 (1.6%)
CCI	Mean ± SD	0.39 ± 0.82	0.29 ± 0.68
	1	912 (14.8%)	3023 (12.3%)
	2	396 (6.4%)	1299 (5.3%)
	≥3	189 (3.1%)	408 (1.7%)
Smoking	Never smoked	5059 (82.3%)	20,248 (82.4%)
	Ever smoked	933 (15.2%)	3245 (13.2%)
	Missing	154 (2.5%)	1079 (4.4%)

All *p* values are <0.05, except for diabetes and COPD. IBD, inflammatory bowel disease; IBS, irritable bowel syndrome; CVD, cardiovascular disease; CKD, chronic kidney disease; CCI, Deyo–Charlson comorbidity index.

**Table 3:** Annual healthcare resource utilization of women diagnosed with endometriosis compared to matched controls.

Annual Healthcare Resource Utilization		Endometriosis (*n* = 6146)	Controls (*n* = 24,572)	Adj. OR (95%CI) *
Visits to gynecologist	≥1 visit	68.1%	55.5%	**1.7 (1.6–1.8)**
	≥5 visits	19.3%	13.2%	**1.6 (1.5–1.7)**
	Median (IQR)	1 (0–4)	1 (0–3)	
Visits to family physician or pediatrician	≥1 visit	94.8%	89.6%	**1.9 (1.7–2.2)**
	≥5 visits	67.5%	53.3%	**1.8 (1.7–1.9)**
	Median (IQR)	7 (3–12)	5 (2–10)	
Hospitalizations	≥1	12.5%	6.0%	**2.3 (2.1–2.5)**
	≥2	3.0%	1.0%	**3.2 (2.6–3.9)**
ER admissions	≥1	8.1%	4.9%	**1.7 (1.5–1.9)**
	≥2	1.9%	1.0%	**1.9 (1.5–2.3)**
Insertion of hormone-releasing IUD		1.2%	1.2%	0.9 (0.7–1.2)
Oral contraceptives	Any	23.6%	15.6%	**1.8 (1.6–1.9)**
	Progesterone-only	4.8%	2.8%	**1.8 (1.5–2.0)**
Hormonal med.	Gonadotropins	4.9%	1.5%	**3.2 (2.8–3.8)**
Pain medication	Cox-2 inhibitors	3.2%	2.1%	**1.6 (1.4–1.9)**
	NSAIDs	9.7%	6.5%	**1.6 (1.4–1.8)**
	PAR, ASA, or dipyrone	7.8%	6.0%	**1.4 (1.2–1.5)**
	Codeine with PAR or ASA	3.4%	2.3%	**1.6 (1.3–1.8)**
	Tramadol	0.9%	0.4%	**2.3 (1.6–3.2)**
Antidepressants	Any type	11.8%	8.6%	**1.4 (1.3–1.5)**
Imaging	MRI	0.6%	0.1%	**10.3 (5.6–19.0)**
	TVUS/pelvic/genital US	41.1%	30.4%	**1.6 (1.5–1.7)**
	(>1 US)	(15.0%)	(7.7%)	
	E-TVUS	3.1%	0.0% ^†^	**29.5 (19.4–44.8)**
Laboratory testing	LH or FSH	16.4%	9.3%	**1.9 (1.7–2.0)**
	CA-125	10.9%	2.0%	**5.9 (5.2–6.7)**
	CA-15.3	2.9%	0.9%	**3.2 (2.6–3.9)**
	CBC	73.2%	62.2%	**1.6 (1.5–1.7)**
	HG ≤ 11	50.4%	43.4%	**1.3 (1.2–1.4)**
	Iron	31.2%	24.5%	**1.4 (1.3–1.5)**

ER, emergency room; IUD, intrauterine device; NSAID, nonsteroidal anti-inflammatory drug; PAR, paracetamol; ASA, acetylsalicylic acid; SSRI, selective serotonin reuptake inhibitor; LH, luteinizing hormone; FSH, follicle-stimulating hormone; CA-125, cancer antigen 125; MRI, magnetic resonance imaging; US, ultrasound; TVUS, transvaginal US; E-TVUS, dedicated endometriosis TVUS; SES, socioeconomic status; BMI, body mass index; HG, Hemoglobin ≤11 at least once within 5y; IQR; interquartile range. * OR from conditional logistic regression, adjusted for SES and BMI. † Includes one patient whose endometriosis diagnosis was confirmed by US in free text only. **Boldface indicates statistical significance (*p* < 0.05).**
